# MTH1 Substrate Recognition—An Example of Specific Promiscuity

**DOI:** 10.1371/journal.pone.0151154

**Published:** 2016-03-21

**Authors:** J. Willem M. Nissink, Michal Bista, Jason Breed, Nikki Carter, Kevin Embrey, Jonathan Read, Jon J. Winter-Holt

**Affiliations:** 1 Chemistry, Oncology, Innovative Medicines and Early Development Biotech Unit, AstraZeneca, Unit 310 (Darwin Building), Cambridge Science Park, Milton Road, Cambridge, CB4 0WG, United Kingdom, and Alderley Park, Cheshire, SK10 4TG, United Kingdom; 2 Discovery Sciences, Innovative Medicines and Early Development Biotech Unit, AstraZeneca, Unit 310 (Darwin Building), Cambridge Science Park, Milton Road, Cambridge, CB4 0WG, United Kingdom; 3 Discovery Sciences, Innovative Medicines and Early Development Biotech Unit, AstraZeneca, Alderley Park, Macclesfield, SK10 4TG, United Kingdom; George Washington University, UNITED STATES

## Abstract

MTH1 (NUDT1) is an oncologic target involved in the prevention of DNA damage. We investigate the way MTH1 recognises its substrates and present substrate-bound structures of MTH1 for 8-oxo-dGTP and 8-oxo-rATP as examples of novel strong and weak binding substrate motifs. Investigation of a small set of purine-like fragments using 2D NMR resulted in identification of a fragment with weak potency. The protein-ligand X-Ray structure of this fragment provides insight into the role of water molecules in substrate selectivity. Wider fragment screening by NMR resulted in three new protein structures exhibiting alternative binding configurations to the key Asp-Asp recognition element of the protein. These inhibitor binding modes demonstrate that MTH1 employs an intricate yet promiscuous mechanism of substrate anchoring through its Asp-Asp pharmacophore. The structures suggest that water-mediated interactions convey selectivity towards oxidized substrates over their non-oxidised counterparts, in particular by stabilization of a water molecule in a hydrophobic environment through hydrogen bonding. These findings may be useful in the design of inhibitors of MTH1.

## Introduction

Nudix hydrolases are phosphohydrolases with a wide range of substrates, which generally take the form nucleoside-diphosphate-X. The products of the hydrolysis reaction are mono-phosphate nucleotides or derivatives. The Nudix pyrophosphatase MTH1 or human MutT-homolog 1 (Nudix-type motif 1, NUDT1) is a DNA-damage-preventing enzyme. It recognizes and disables oxidized nucleotides through removal of a pyrophosphate from the damaged nucleotide [1,2], which subsequently prevents their incorporation in DNA or RNA. Free nucleotides are approximately 13,000 times more susceptible to oxidation compared with nucleic bases that are incorporated in DNA or RNA [3] and incorporation of such damaged nucleic bases can ultimately lead to point mutations. The enzyme is reported to recognize a range of substrates including 2-hydroxy-dATP, 2-hydroxy-rATP, 8-oxo-dGTP, and 8-oxo-dATP [4–6].

Cancer cells often exhibit a higher level of oxidative stress than is seen in normal tissue as a result of changes in metabolic pathways, leading to elevated levels of oxidised nucleotides. MTH1 is hypothesized to be essential for the survival of tumour cells [7–12] though reports to the contrary can also be found [13]. Inhibition of MTH1 has been proposed to cause elevated incorporation of oxidized bases in RNA or DNA, and thereby elevated levels of mutagenic stress in cancer cells, leading to cell death. [14,15].

In this paper we explore the mechanism by which MTH1 recognises oxidized nucleotides over their non-oxidised counterparts, and we present protein-substrate structures of MTH1 with 8-oxo-dGTP and 8-oxo-rATP, as well as structures of MTH1 ligands identified in fragment screens (Table 1). These were characterised structurally to shed light on the motifs that bind the central Asp-Asp anchor in MTH1, and may suggest novel ways of inhibiting this oncology target.

**Table 1:** Data collection and structure refinement statistics for MTH1 complexes. **Numbers in brackets indicate statistics for the highest resolution shells**.

PDB Code	5FSI	5FSK	5FSL	5FSM	5FSN	5FSO
Compound in paper	*8-oxo-dGTP*	*8-oxo-rATP*	3	5	6	7
Space group:	P21212	P21212	P21212	P21212	P21212	P21212
	60.4690	60.4950	60.3180	60.2130	60.3600	60.3130
Cell constants: a b c (Å),	66.1490	65.6085	65.9260	66.1990	66.2170	66.1430
	36.0830	36.3480	35.9990	36.3120	36.2230	36.1970
Resolution range (Å)	44.63–1.63	60.5–1.56	60.32–1.24	66.2–1.67	60.36–1.69	66.14–1.67
Highest resolution shell (Å)	1.71–1.63	1.75–1.56	1.33–1.24	1.76–1.67	1.80–1.69	1.76–1.67
Completeness overall (%)	99.1 (96.3)	99.6 (98.7)	86.3 (48.1)	84.0 (26.8)	82.5 (31.1)	85.2 (31.7)
Total number of observations	119037	242361	208155	82821	85405	84353
Reflections, unique	18663	21084	35440	14813	13844	14865
Redundancy	6.4 (6.1)	11.5 (8.6)	5.9 (3.6)	5.6 (1.7)	6.2 (2.9)	5.7 (1.8)
*R*merge _overall_ ^1^	0.161 (0.77)	0.066 (0.585)	0.04 (0.46)	0.04 (0.148)	0.055 (0.386)	0.034 (0.101)
I/SigI^2^	11.6 (5)	21 (3.3)	22.1 (2.4)	28.9 (4.3)	23.4 (2.8)	35.4 (6.6)
*R*value _overall_ (%)^3^	20.1	20.4	20.2	18.0	18.8	15.2
*R*value _free_ (%)^4^	22.5	24.2	21.1	21.0	22.5	22.9
Non hydrogen protein atoms	1221	1237	1222	1240	1245	1252
Non hydrogen ligand atoms	32	32	13	14	13	10
Solvent molecules	51	74	104	176	158	164
R.m.s. deviations from ideal values						
Bond lengths (Å)	0.01	0.01	0.01	0.01	0.01	0.01
Bond angles (°)	1.09	1.11	1.05	1.06	1.07	1.05
Average B values (Å^2^)						
Protein main chain atoms	17.2	20.3	13.2	12.5	14.0	12.3
Protein all atoms	19.9	22.5	14.9	17.8	17.2	15.2
Ligand	23.6	31.6	15.5	18.1	20.1	8.1
Solvent	29.1	35.3	26.7	31.2	33.0	31.1
Φ, Ψ angle distribution for residues ^5^						
In most favoured regions (%)	92.3	93.8	93.1	94.6	93.1	94.6
In additional allowed regions (%)	7.7	6.2	6.9	5.4	6.9	5.4
In generously regions (%)	0	0	0	0	0	0
In disallowed regions (%)	0	0	0	0	0	0

^**1**^ *R*_merge_ = Σ_*hkl*_ [(Σ_*i*_ |*I*_*i*_ - ‹*I*›|) / Σ_*i*_ *I*_*i*_]

^**2**^ I/sigI avg is the mean I/sig for the unique reflections in the output file

^**3**^ *R*_value_ = Σ_*hkl*_ ||*F*_obs_|—|*F*_calc_|| / Σ_*hkl*_ |*F*_obs_|

^**4**^ *R*_free_ is the cross-validation *R* factor computed for the test set of 5% of unique reflections

^**5**^ Ramachandran statistics as defined by PROCHECK^10^

## Materials and Methods

### Protein expression and purification

Full length p18 isoform MTH1 (M1-156) with an N-terminal 6-His TEV protease cleavable tag was obtained from the Structural Genomics Consortium (SGC) (PDBID: SGC:3Q93) and expressed in E. coli Gold BL21(DE3) cells. Cultures were grown in Terrific Broth (TB) media in the presence of Kanamycin (100 μg/ml) and Tetracyclin (12.5 μg/ml). Cells were initially grown at 37°C and when OD600 0.6 was reached; cells were cooled to 18°C for 20 hours and then harvested by centrifugation at 12,000 x g. Cells were re-suspended in Lysis buffer (40 mM HEPES pH8.0, 300 mM NaCl, 20 mM Imidazole, 1 mM TCEP, 1 mg/ml lysozyme, and 1/10,000 dilution benzonase) supplemented with EDTA free Complete Protease inhibitors (Roche). Cells were lysed using a cell disruptor (Constant Systems TS Series). The lysate was centrifuged at 35,000 x g for 60 mins using a JLA-16.250 Beckman rotor. The supernatant was loaded onto 10 mls of NiNTA resin which had been washed with 100 ml of Equilibration buffer (40 mM HEPES pH 8.0, 300 mM NaCl, 20 mM Imidazole, 1 mM TCEP). The resin was then washed with 100 ml of equilibration buffer and 3 x 10 ml of wash buffer (40 mM HEPES pH 8.0, 300 mM NaCl, 40 mM imidazole, and 1 mM TCEP). MTH1 was eluted using 3 x 10ml of elution buffer (40 mM HEPES pH 8.0, 300 mM NaCl, 500 mM imidazole, 1 mM TCEP). Recombinant TEV (Tobacco Etch Virus) protease was then used to remove the N-terminal 6-His tag during an overnight dialysis step at 4°C vs. 40 mM HEPES pH 8.0, 300 mM NaCl, 10 mM imidazole, 1 mM TCEP. The dialysed protein was re-passed over a 3.5 ml gravity-flow NiNTA column at 4°C. The unbound fraction was concentrated to 5 ml using a 10 K MWCO centrifugal concentrator. This 5 ml sample was loaded onto a 120 ml S75 column which had been equilibrated in 20 mM Tris pH 7.4, 150 mM NaCl, 5% glycerol, and 2 mM TCEP. The protein was concentrated using a 10 K MWCO centrifugal concentrator at 4°C to ~41 mg/ml and snap frozen using liquid nitrogen in 50 μl aliquots.

### Chemistry

Compounds **1**–**7** are available from commercial vendors. Substrates were purchased from vendors as indicated in the sections below.

### Substrate K_m_ determination

MTH1 substrate K_m_ determination assay conditions were 0.2 nM MTH1 and a concentration range of substrate from 0–300 μM, diluted in MTH1 reaction buffer (100 mM HEPES pH 7.5, 10 mM MgCl, 1 mM DTT and 0.005% Tween 20). After a reaction time of 15 minutes, pyrophosphate (PPi) released by the reaction was detected using the PPiLight inorganic pyrophosphate assay kit from Lonza and luminescence was monitored using the Perkin Elmer EnVision multimode plate reader. Substrates dGTP and 2-OH—dATP were sourced from Jena Bioscience. Substrates 8-oxo-dGTP, 8-oxo-rGTP, 8-oxo-dATP, 8-oxo-rATP and 2-OH-rATP were sourced from TriLink BioTechnologies.

All oxidised ATP-derived substrates were then tested using an assay format based on the enzymatic hydrolysis of the nucleotide substrate by purified human recombinant MTH1 to form the corresponding monophosphate nucleotide and pyrophosphate. An excess of inorganic pyrophosphatase was added to the assay, which allows the quantification of released inorganic phosphate as a measure of product levels in a coupled enzymatic assay. Inorganic phosphate was measured using an absorbance assay based on malachite green as described by Baykov *et al*. [16]. The assay buffer in which MTH1, inorganic pyrophosphatase and substrates were diluted consisted of 100mM Tris at pH 7.4, 40 mM NaCl, 10 mM MgCl, 0.005% Tween 20 and 1 mM DTT. The final conditions in the assay during enzymatic incubation were 0.2 nM recombinant human MTH1, a three fold dilution range of 0–300 μM oxidised nucleotide substrate and 0.2U per ml inorganic pyrophosphatase.

### 2D-NMR

Uniformly ^15^N-labeled MTH1 was prepared as above with the TB media replaced M9 minimal media supplemented with 5 g/l of IsoGro base powder (^15^N, 98%, Isotec) and 2 g/l of glucose. NMR samples were 100 μM in 50mM HEPES pH 7.4, 50 mM NaCl, 1 mM TCEP, 0.1 mM EDTA, 0.02% NaN_3_ and employing 1% d^6^-DMSO as a lock signal. Spectra were acquired at 298 K on a Bruker Avance 600 MHz spectrometer running Topspin 2.1, equipped with a 5mm TCI cryoprobe with Z-axis gradients. The protein was titrated with compound stocks (100 mM in d6-DMSO) to give typical final compound concentrations 0, 50, 100, 198, 396, 593, 984, 1949 and 3824 μM; these were measured using ^1^H-^15^N-SOFAST-HMQC [17] experiments recorded with 1024x50 (t_2_,t_1_) complex points (in States-TPPI mode), and 9615x1624 Hz (^1^H,^15^N) spectral widths, (53.4 ms x 30.8 ms) acquisition times, respectively). The affinity of the compounds was determined via simultaneous nonlinear fitting of fast-exchange chemical shift perturbations (typically 5 amide correlations, employing absolute value of the linear ^1^H,^15^N chemical shift vector), with the GraFit package [18] against compound concentration using a mass action binding isotherm equation.

### Surface plasmon resonance (SPR)

SPR experiments were performed using a Biacore T200 biosensor (GE Healthcare). Series S NTA sensor chips (GE Healthcare) were used. All experiments were carried out using assay buffer containing; 50 mM HEPES pH 7.5, 150 mM NaCl, 0.005% Tween-20 (v/v) and 1% DMSO (v/v) as running buffer. Typically, 10 mM DMSO stocks of compounds were diluted 1:100 (v/v) in 50 mM HEPES pH 7.5, 150 mM NaCl, 0.005% Tween-20 (v/v) to form 100 μM intermediate stocks with final DMSO concentration of 1% (v/v). The intermediate solutions were then subsequently diluted using assay buffer to achieve final concentration range.

Histidine-tagged MTH1 (isoform p18 (M1-V156)) was immobilized as the ligand onto NTA sensor chips using a capture coupling method [19]. Assay buffer was used as immobilization buffer. The nitrilotriacetic (NTA) surface was first activated with a 2-min injection of 500 μM NiCl_2_ in running buffer before the carboxymethyl dextran surface was activated with a 7-min injection of a 1:1 ratio of 0.4 M EDC and 0.1 M NHS. His-tagged protein was diluted into running buffer to a concentration of 20 μg/mL and immobilized to the surface with a 7-min injection. Remaining activated groups were blocked with two consecutive 60-sec pulses of 0.5 M Ethanolamine. Typical immobilization levels ranged from 500 to 3000 resonance units (RU).

Biacore T200 Evaluation software (GE Healthcare) was used for data processing and analysis. Equilibrium binding analysis was analyzed by plotting steady-state response level vs. compound concentration and fitting to the Langmuir equation (describing simple 1:1 binary interaction).

### Protein crystallization, data collection and structure solution

For compounds **3**, **5**, **6 7** and 8-oxo-rATP, MTH1 protein was crystallized using the hanging drop method at 20°C using 2 μL well solution containing 25% (w/v) PEG3350, 200 mM lithium sulphate and 100 mM sodium acetate pH 4.5 mixed with 2 μL protein solution. Protein crystals were transferred to a solution containing 27% (w/v) PEG3350 200 mM lithium sulphate 100 mM sodium acetate pH 4.5 20% (v/v) DMSO and 2 mM compound. After 20 hours, crystals were removed from the soaking solution and frozen directly in a stream of nitrogen at a temperature of 100 K.

For 8-oxo-dGTP, MTH1 protein was crystallized using the hanging drop method at 20°C using 2 μL well solution containing 25% (w/v) PEG3350, 200 mM lithium sulphate and 100 mM PCTP pH 4.5 mixed with 2 μL protein solution. Protein crystals were transferred to a solution containing 27% (w/v) PEG3350 200 mM lithium sulphate 100 mM PCTP pH 4.5 20% (v/v) DMSO and 2 mM compound. After 20 hours, crystals were removed from the soaking solution and frozen directly in a stream of nitrogen at a temperature of 100 K.

### Data collection

Diffraction data for MTH1 complex with X3, 8-oxo-dGTP and 8-oxo-rATP were collected at the Diamond beamline I03 with an Si111 monochromator with the beam focused by a pair of Kirkpatrick-Baez mirrors on a Dectris Pilatus 6M using a wavelength of 0.97625 Å. Diffraction data were for MTH1complex with X5, X6 and X7 were collected on a Rigaku FRe+ equipped with a Saturn 944 CCD X-ray detector, using Nickel filtered CuKα wavelength of 1.5418 Å.

### Structure solution and refinement

For MTH1 complexes with compounds **3**, **5**, **6**, **7**, 8-oxo-rATP, and 8-oxo-dGTP data were processed using MOSFLM and AIMLESS and reduced using CCP4 [20]. The structures were solved by molecular replacement using coordinates of MTH1 (PDB entry 4N1T) as a template model using CCP4 software. X-ray refinement restraints were generated with Grade and refined with MOGUL. Protein and inhibitor were modeled into the electron density using COOT [21]. The models were initially refined using REFMAC5 [22] and completed with BUSTER [23].

Data collection and protein structure refinement statistics for all MTH1 complexes are shown in Table 1. An example electron density map is shown in Fig B in S1 File.

## Results and Discussion

### Substrates of MTH1

MTH1 recognises multiple oxidized-nucleotide substrates. Most commonly, 8-oxo-dGTP and 2-OH-dATP have been put forward in the literature as substrates for MTH1 [4–6]. Table 2 shows turnover rates (K_m_) for a number of substrates. Our resulting K_m_ values of 11.3 μM for 8-oxo-dGTP; 7.6 μM for 8-oxo-dATP; 14 μM for 2-OH-dATP; and 13.4 μM for 2-OH-rATP are in good agreement with published values and suggest that these substrates are processed by MTH1 with similar turnover rates. The assay we used employed a pyrophosphate detection kit that utilized ATP to generate a readout, and inhibition of MTH1 was observed as a result of the presence of ATP. It is known that ATP, ADP, and other non-oxidised nucleotides are weak binders competing with the natural substrates but are not themselves dephosphorylated [5]. Given its abundance in cells at up to millimolar levels [24], a weak binder like ATP is expected to compete with native substrates of MTH1 in cells. The observation that ATP inhibits MTH1 is unsurprising in light of the observation by Gad et al. that (*S*)-crizotinib inhibits MTH1 [7]. This compound is the enantiomer of a known kinase inhibitor (*R*)-crizotinib, and itself an ATP mimic that is capable of binding in a kinase ATP site.

**Table 2:** Subset of MTH1 substrates with K_m_ values from experiment and as reported in the literature.

*Substrate*	*K*_*m*_ (μM)*	
dGTP	4.2x10^2^±1.9x10^2^	(this publication)
	258	[4,34]
8-oxo-dGTP	11±2.7	(this publication)
	13.2±2.5	[6]
	15.2	[35,36]
	17.3	[4,34]
8-oxo-rGTP	82±45	(this publication)
	55	[4,34]
8-oxo-dATP	7.6±0.50	(this publication)
	13.9	[26]
	15.2	[4,34]
8-oxo-rATP	2.4x10^2^±94	(this publication)
	51	[4,34]
2-OH-dATP	14±6.0	(this publication)
	8.3	[4,34]
2-OH-rATP	13±2.5	(this publication)
	4.3	[4,34]

* error provided where available

### The Asp-Asp unit in the MTH1 binding site is a key binding motif

The Nudix hydrolase family covers 24 human genes [2]. Their commonality lies in the Nudix hydrolase motif, which covers the catalytic site. The substrate recognition site is adjacent to and mostly separate from the catalytic site and in MTH1 it contains a hydrogen-bonding recognition element in the form of an Asp-Asp motif. This motif is unique to MTH1 within the set of human Nudix hydrolases and is important for substrate recognition.

**Fig 1:**
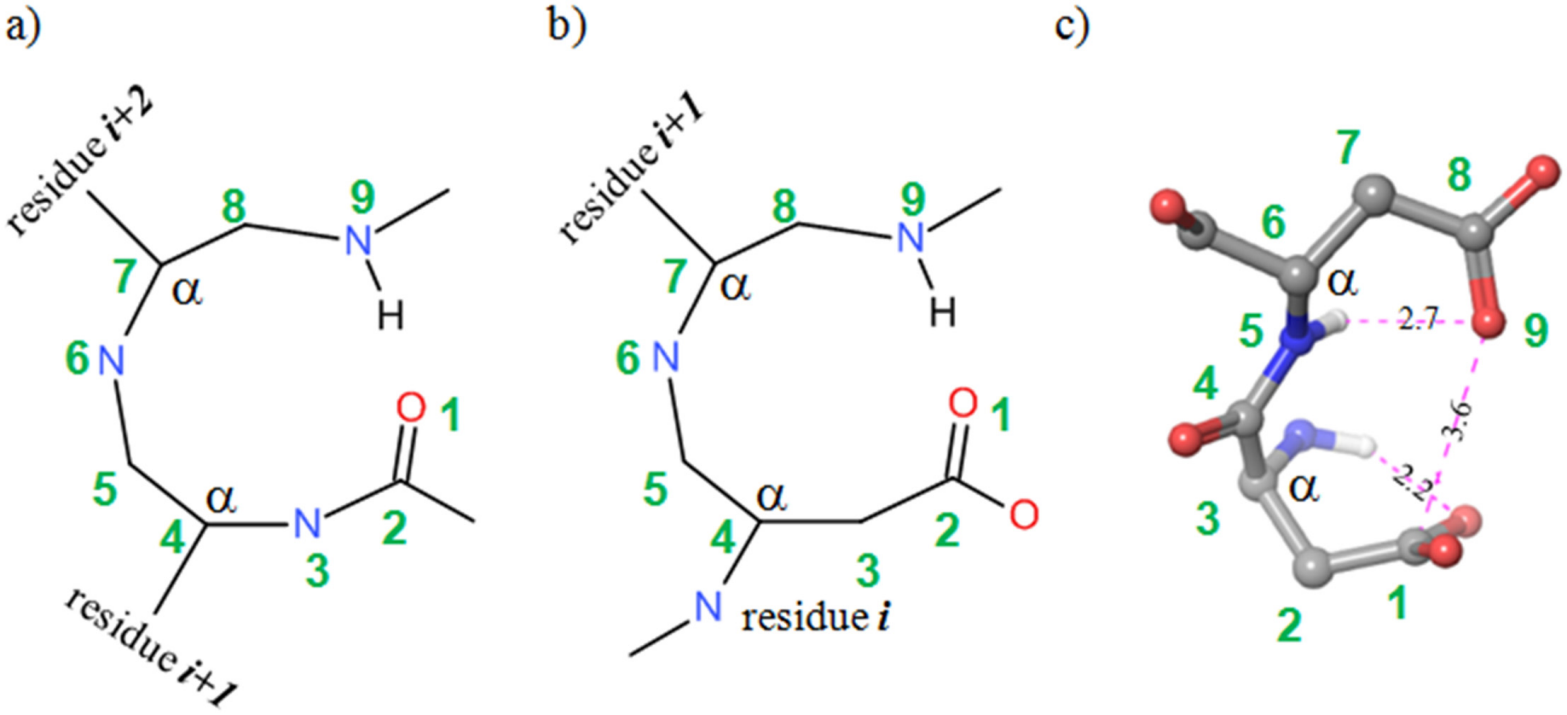
a,b) Nine-membered cycles in beta-turns (a) and Asx (b) turn [25]. c) Asp –Asp motif in MTH1 structure. Numbering and α-carbon indication added for the purpose of comparison.

**Fig 2:**
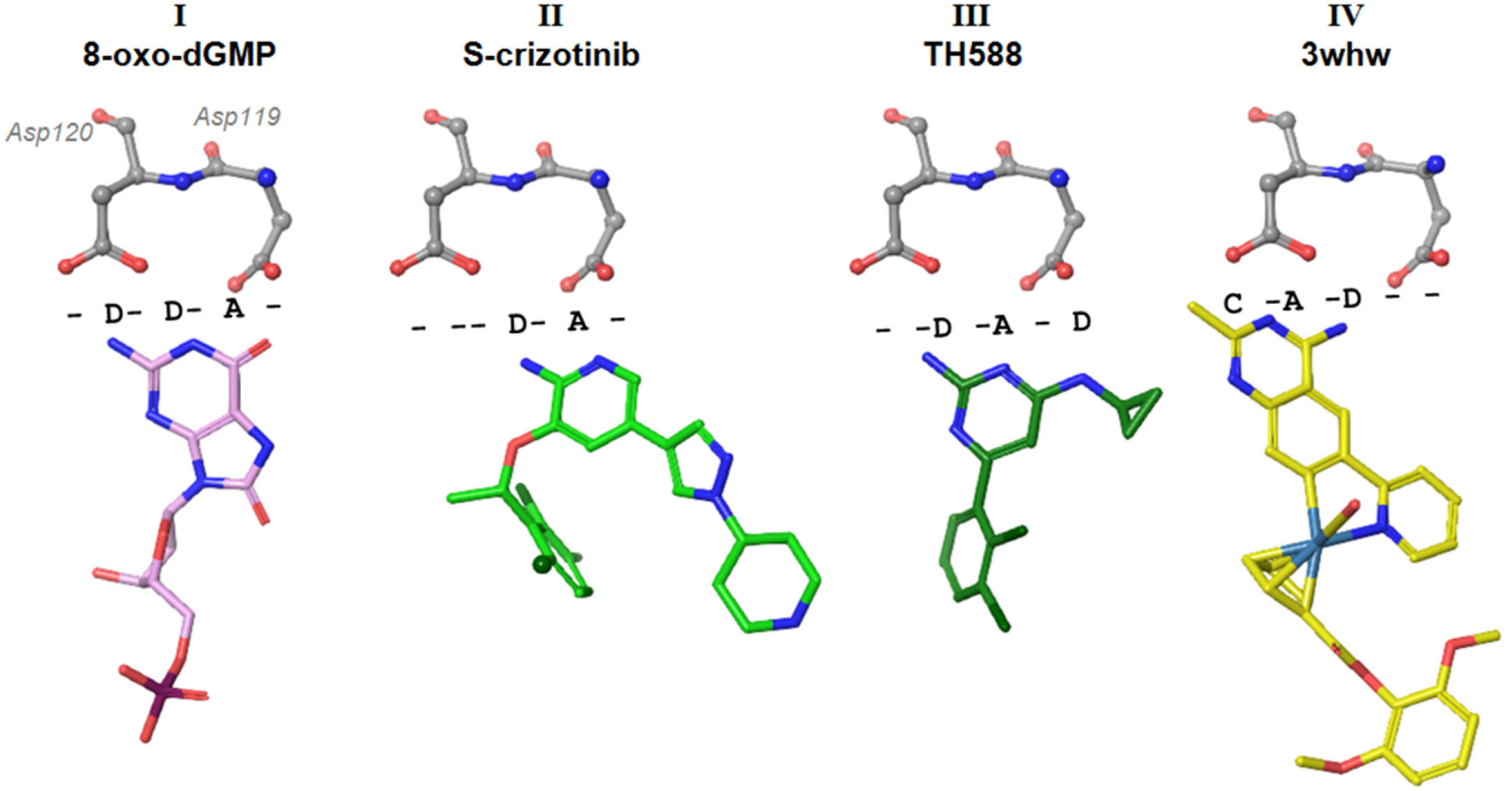
Binding modes observed in published structures of MTH1 with I): product 8-oxo-dGMP [6] (PDB 3ZR0) and inhibitors II) aminopyrimidine (S)-crizotinib [8] (PDB 4C9X) III) bis-aminopyrimidine TH588 [7] (PDB 4N1U), and IV) an organometal methylaminopyrimidine inhibitor [26] (PDB 3WHW). The spatial contact motif is summarised in a textual annotation using ‘D’ (donor), ‘A’ (acceptor), ‘C’ (carbon, lipophilic) and ‘-‘ (no near contact) describing the space between protein and ligand. Anchoring motif Asp120-Asp119 is shown for reference.

The Asp-Asp motif is reminiscent of beta-turns and Asx-turns described in ref. [25]. Its nine-membered ring conformation is stabilized by intramolecular hydrogen bonds and carbonyl-carbonyl contacts (Fig 1a, 1b and 1c). One of the oxygen atoms of D119 serves as an acceptor in a hydrogen bond with the indole NH of W123. As a result the Asp-Asp motif is rigid, and is an anchoring point for the binding of substrates like 8-oxo-dGTP (Fig 2, binding mode **I**), via the nucleobase.

All substrates form strong hydrogen bonds to the Asp-Asp motif (distances range from 2.4 to 3 Å), but not necessarily to both residues. Sakai et al. [5] showed that mutations of D119 to A or N did not affect 8-oxo-dGTP recognition, and suggested that D120 is the key element for binding. Residue D119 was shown to be essential for binding 2-OH-dATP, suggesting that interactions to both of the aspartate residues or D119 alone can support binding of a substrate.

Several ligand-bound structures of MTH1 are available in the public domain (Fig 2, motifs **II** [8], **III** [7], **IV** [26] and all show interactions with D119 (**II**), D120 (**IV**) or both (**I** and **III**). Substantial variation in placement of the Asp-Asp binding moiety is observed across the binding modes of MTH1 inhibitors, suggesting that while the Asp-Asp motif functions as a key recognition and anchoring element, the hydrogen-bonded motif of the ligand can nonetheless be seen to shift along the Asp-Asp axis (Fig 2). The protonation states of the Asp-Asp motif are restricted by its intramolecular interaction between the carbonyl groups (hindering protonation), and intramolecular hydrogen-bonding to the indole NH of W123 to one of the carboxylate oxygens of D120. The acceptor pattern seen for binding modes **I** and **II** (Fig 2, substrate and (*S*)-crizotinib) suggest that the carboxylic oxygen of D119 may be protonated. The donor-acceptor pattern of substrate 8-oxo-dGTP in binding mode **I** suggests that the D120 carboxylate remains unprotonated in this case.

A different donor/acceptor pattern is observed in binding modes **III** and **IV** (see Fig 2). Binding mode **III**, with ligand TH588, introduces an acceptor and donor in the region near Asp119 where the 8-oxo-dGTP substrate presents opposite groups. Binding mode **IV** shifts this motif, such that its main interactions are with D120. The reported IC_50_ inhibitory potencies of the ligands shown are: (*S*)-crizotinib, 72 nM; TH588, 5 nM; 3whw, 6 nM. These observations suggest that the Asp-Asp motif can interact with substrates with superficially similar donor/acceptor motifs in quite different ways. It further implies that, whilst these interactions are important for binding potency, explicit recognition is controlled by other factors—a consequence of which is the substrate promiscuity for this target (Table 2).

### Hydrogen bonding pattern, water-, and Met81 interactions enable substrate selectivity

Given the plasticity of the Asp-Asp hydrogen-bonding networks it is not immediately clear how this would support substrate selectivity. Svensson et al. [6] suggest that the 8-oxo-dGMP product binds to the Asp-Asp motif through a 1N-6OH tautomer (enol form) of the base, while non-oxidised dGMP binds only very weakly. This may be corroborated by the observation that the D119A mutation retains 8-oxo-dGTP binding [5]. However, the question remains why the 1N acceptor of such an enol is placed near the central carbonyl of the Asp-Asp motif. The observation of an acceptor in this position is not in line with the hydrogen bonding patterns seen in various inhibitor structures (Fig 2, binding modes **II**, **III**, **IV**). Protonation of the relevant aspartate would provide a hydrogen bond to the N1 acceptor, but destabilize the carbonyl-carbonyl contact of the Asp-Asp arrangement (Fig 1c, protonation of atom 9). The short 2.4 Å distance of the oxygen of Asp119 to 8-oxo-dGMP would be explained equally well by protonation of the aspartate oxygen and binding to the keto tautomer.

The pK_a_ of an aspartate residue in solution is typically 3.9, but burial of such residues in a binding pocket is known to alter acidity [27]. Estimation of the acidity of D119 and D120 in the MTH1 nucleotide pocket suggests that their pKa values lie around 6, suggesting that either can be protonated at physiological pH [27,28]. Earlier publications [29–32] and calculation of pKa for guanine and 8-oxoguanine model compounds (Fig A in S1 File) suggest that, with regard to recognition of the Asp-Asp motif, the ‘keto’ form is likely. N1 will be protonated as a result, both for a guanine base and its oxidised 8-oxo variant. This would be consistent with the donor-acceptor patterns observed for various inhibitors, as shown in Fig 2.

**Fig 3:**
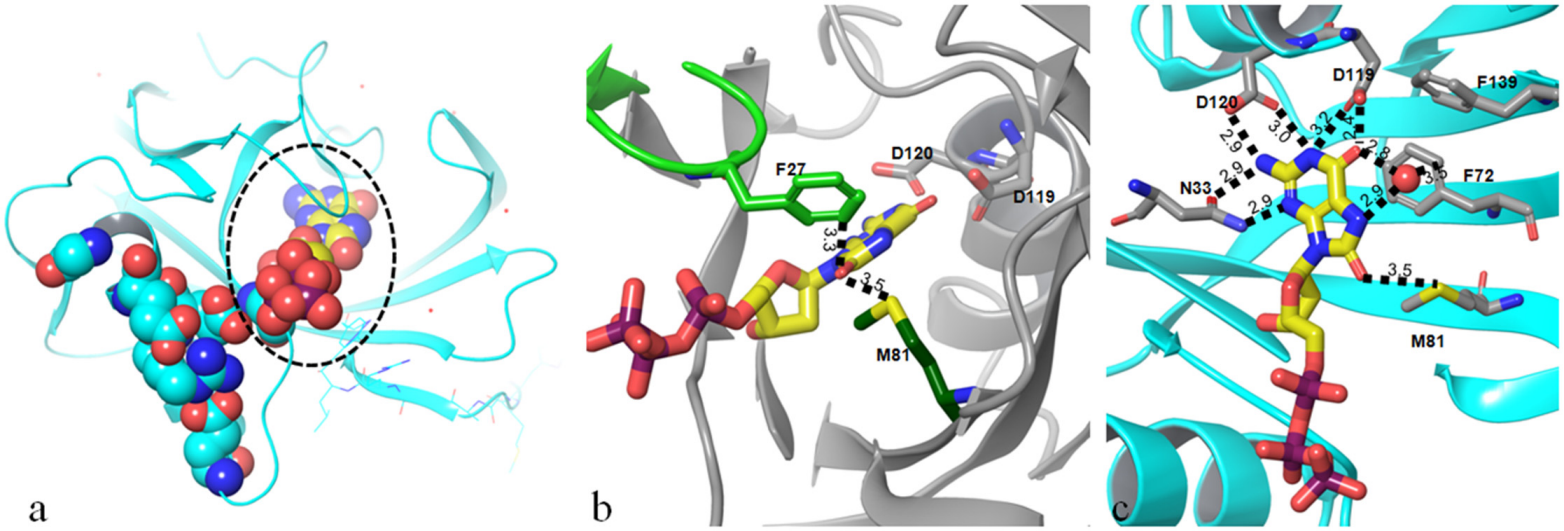
8-oxo-dGTP structure. a) Nudix-box motif (cyan spacefilled residues) shown in relation to the substrate binding site (circled); substrate 8-oxo-dGTP is shown in yellow. b) Non-hydrogenbonding contacts of the 8-oxo group of 8-oxo-dGTP to Phe27 in loop (light green) and Met81 (dark green). c) Hydrogen bonding and contact pattern around 8-oxo-dGTP. Red sphere indicates water molecule. Distances indicated in Ångstrom.

While there does not seem to be evidence for a mechanism of recognition dependent on the head group, several other factors may contribute to selective recognition of the oxidized nucleotide. Firstly, in our structure of the triphosphate 8-oxo-dGTP (Fig 3a), the sulfur of the Met81 side chain is in close contact with the 8-oxo carbonyl group (3.5 Å) and Phe27 (3.3 Å), suggesting a favourable interaction is absent in non-oxidised compounds which lack the 8-oxo group. This residue sits at the tip of a flexible loop which leads to the catalytic residues, and its organization may be needed to prime the enzyme for catalysis. Secondly, introduction of the keto group into the guanine nucleobase changes the acceptor N at the 7-position to a donor NH in the oxidized compound. This group and the 6-oxo group bind a water tightly (2.9 Å and 2.8 Å distances; Fig 3b), which appears to be positioned in a small and fairly hydrophobic cavity formed by phenyl rings of Phe 27, 72 and 139, and Trp123 (numbering as in PDB structure 3zro [6]). The distance of the water to the ring of Phe 72 is 3.5Å. The short contacts from the nucleobase to this water leave one water hydrogen free to interact with a π-system of nearby rings (Phe72, Phe139, Trp117). The 7-N and 6-oxo groups in the non-oxidised guanine variant would both be acceptors. It would be unlikely for these two acceptors to form both hydrogen bonds with a single water molecule, which would render proximity of the water less favourable in the case of a substrate lacking the 8-oxo group. As such, we propose that the stabilization of this water plays a key role in the selective recognition of 8-oxo-dGTP over non-oxidised dGTP.

### Structural basis for substrate recognition

To investigate the binding motifs for substrates of MTH1 in more detail we produced protein crystal structures for substrates with different K_m_ values: 8-oxo-rATP (K_m_ = 241 μM) and 8-oxo-dGTP (K_m_ = 7.6 μM) (attempts to generate a crystal structure of 2-OH dATP were unsuccessful). Our 8-oxo-dGTP structure (density of substrate shown in Fig B in S1 File) is similar to a published structure of the cleaved mono-phosphate nucleotide product [6]. Interestingly, in both 8-oxo-dGTP and 8-oxo-rATP structures we observe electron density for all phosphate groups. No metal ion is bound to the active site residues (Glu100, Glu56, Glu52) that lie in the Nudix motif (which itself is adjacent to the substrate pocket, Fig 3a), and this is consistent with the absence of substrate cleavage. This is possibly due to the fact that the structures were produced through soaking of apo crystals rather than co-crystallysation, and we hypothesise that the packing of the proteins prevents the catalytic machinery from cleaving the phosphates on the timescale of the experiment. Although occupancy of the terminal pyrophosphate is lower than that of the rest of the molecule, the placement of these groups is clear.

**Fig 4:**
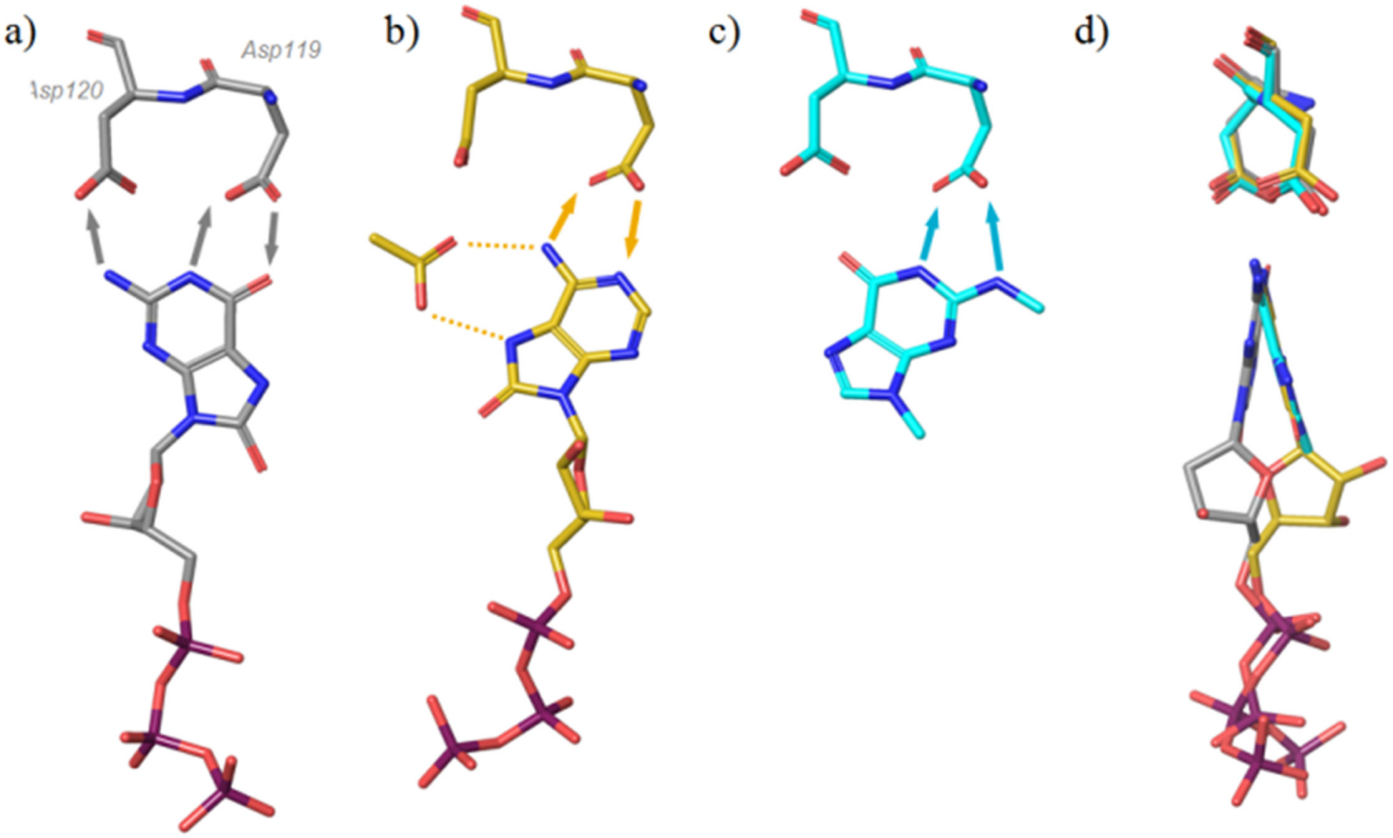
Binding modes of a) 8-oxo-dGTP and b) 8-oxo-rATP c) binding mode fragment 3 d) rotated view of a,b,c from left hand side. Arrows indicate suggested donor-to-acceptor hydrogen bond directions and dotted lines, interaction of acetate.

Fig 4 shows a comparison of the binding modes of (a) 8-oxo-dGTP and (b) 8-oxo-rATP in MTH1. It is apparent that the binding interactions of 8-oxo-dGTP are very different from those seen for 8-oxo-rATP, with the latter engaging Asp119 almost exclusively. The 8-oxo-rATP nucleotide is rotated 180 degrees along its axis compared with the binding mode of 8-oxo-dGTP. As a result, the ribose and deoxyribose sugars of the molecules do not align, but both modes show good alignment of the P1 phosphate. The deoxyribose hydroxyl group forms a hydrogen bond to Thr8 (backbone carbonyl, not shown), but this contact is not present in the case of 8-oxo-dATP, where the ribose hydroxyls point in the opposite direction towards Phe27. The sidechain of Phe27 is not resolved in the structure of 8-oxo-dATP as a result of this steric clash, and it is likely that the destabilization of this loop affects alignment of the catalytic machinery with the substrate. In the 8-oxo-rATP structure the space near Asp120 resulting from this flip in binding mode has been filled with a buffer acetate ion (Fig 4b), which itself forms hydrogen bonds to the purine base.

Mutational studies by Sakai et al. [5] have suggested the necessity of Asp119 for the binding of another substrate, deoxyribose variant 2-OH-dATP, with D119A abrogating turnover of the substrate completely. Such an effect would be consistent with the binding mode of the nucleic base as seen for 8-oxo-rATP (Fig 4b), where Asp120 shows little involvement in substrate binding, and with placement of the ribose hindering turnover of 2-OH-rATP in MTH1.

### Binding of nucleotide-like fragments by NMR and protein crystallography

**Fig 5:**
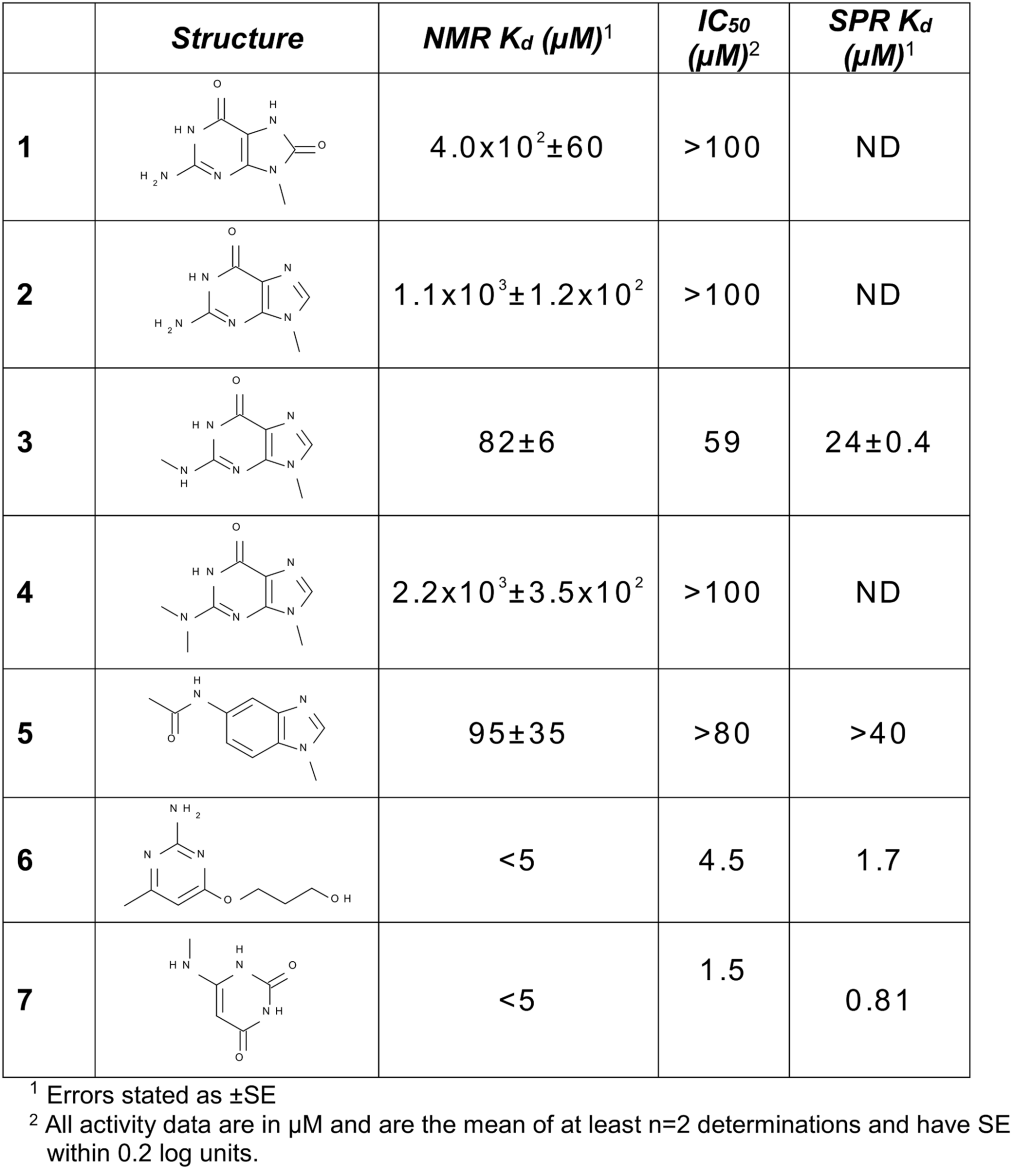
NMR K_d_, biochemical IC_50_, and SPR K_d_ for fragments exploring nucleotide binding pharmacophores.

**Fig 6:**
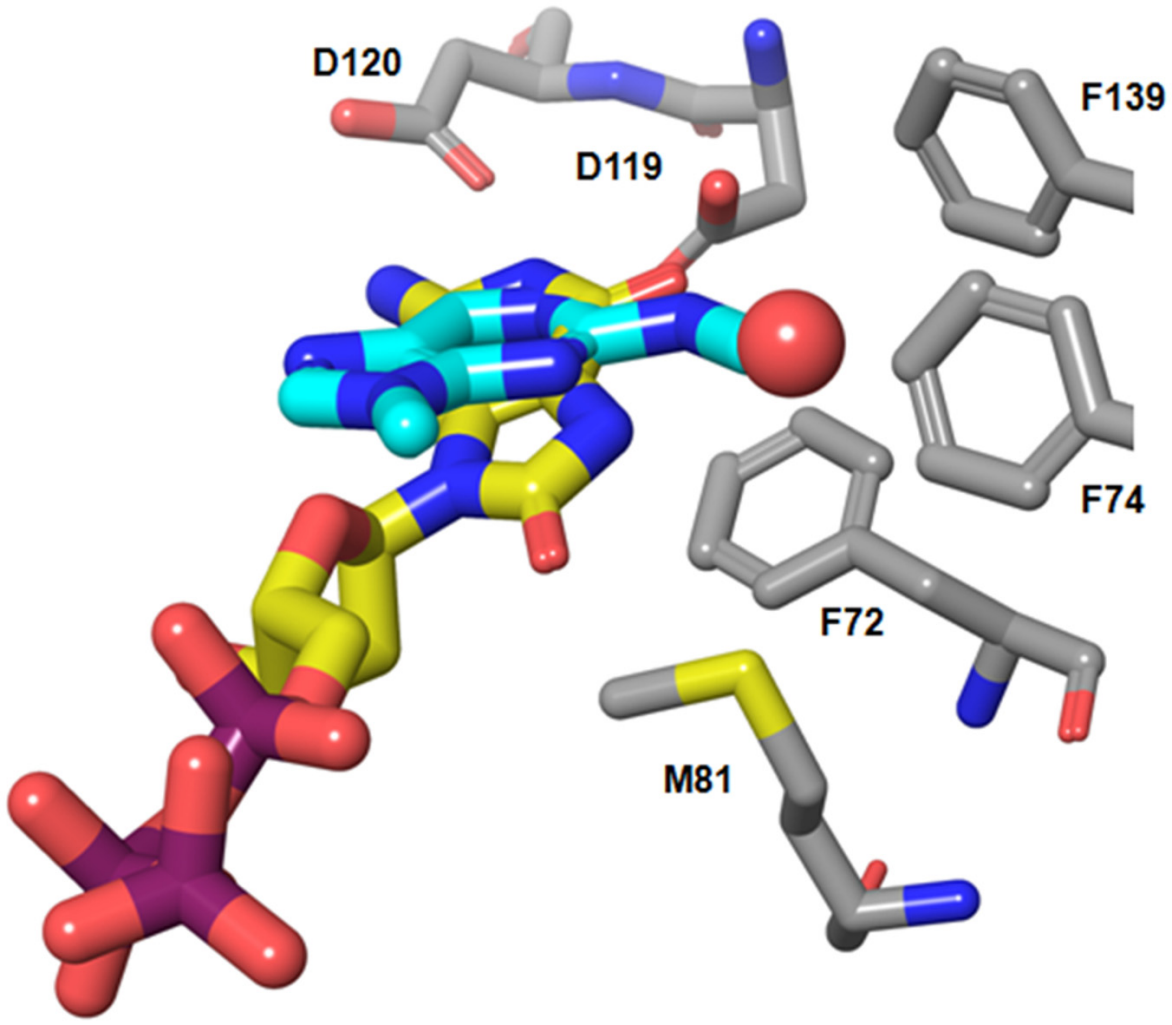
Binding modes of 8-oxo-dGTP (yellow carbons) and 3 (cyan carbons). The water shown is present in the 8-oxo-dGTP structure (see Fig 3b) but is displaced in the fragment structure.

To explore the binding behaviour of different pharmacophores, a small set of nucleotide-like fragments was collated to cover a variety of binding features and purine-like binding motifs. Their binding to MTH1 was determined using ligand-observed NMR (K_d_ values in Fig 5). Compound **1** is an 8-oxo variant that shows an affinity of 400 μM for MTH1. Compound **2** is the des-oxo matched-pair of **1** (ie. a mimic of non-oxidised guanine) and shows a threefold lower affinity than the 8-oxo compound. Fragments **3** and **4** explore methylation of the 2N amino group of the guanine base. A surprising thirteen-fold gain in potency is observed for fragment **3**, a mono-amino-methylated version of des-oxo compound **2.** Dimethylation of the amino group as in **4** and thereby abrogation of its hydrogen-bonding results in a two-fold decrease in potency (increase in K_d_) as derived by NMR compared with the non-methylated amine **2**, which is perhaps less than expected if the amino group was in bonding contact with the Asp-Asp motif. To investigate this, **3** was crystallized with MTH1. Fig 4c shows orientation of **3** with regard to the Asp-Asp motif, and a comparison with the 8-oxo-dGTP substrate placement is shown in Fig 6.

The aromatic system of **3** shows a different orientation of the bicyclic base compared with 8-oxo-dGTP, and its aminomethyl group can be seen to bind Asp119 as well as displace a water molecule from the hydrophobic pocket near Phe74. Its orientation with respect to the Asp-Asp motif is very similar to that seen for TH588 (Fig 2, binding mode **III**), with the difference that the amino group of TH588 is replaced by a keto group.

In the case of the 8-oxo-rATP substrate, the presence of the water in its original location becomes unfavourable, thereby reducing the affinity of the 8-oxo-rATP substrate for MTH1 compared to the 8-oxo-dGTP substrate. However, in the case of the fragment the water is favourably displaced from its hydrophobic surroundings and replaced by a hydrophobic methyl group, resulting in a relatively high affinity for such a small fragment. Again, this suggests that the water plays a role in the recognition of substrates, lowering the affinity of those substrates that do not stabilize it by suitable binding interactions, but enabling binding of groups that displace it with a hydrophobic moiety.

### MTH1 binds different hydrogen-bonding pharmacophores

**Fig 7:**
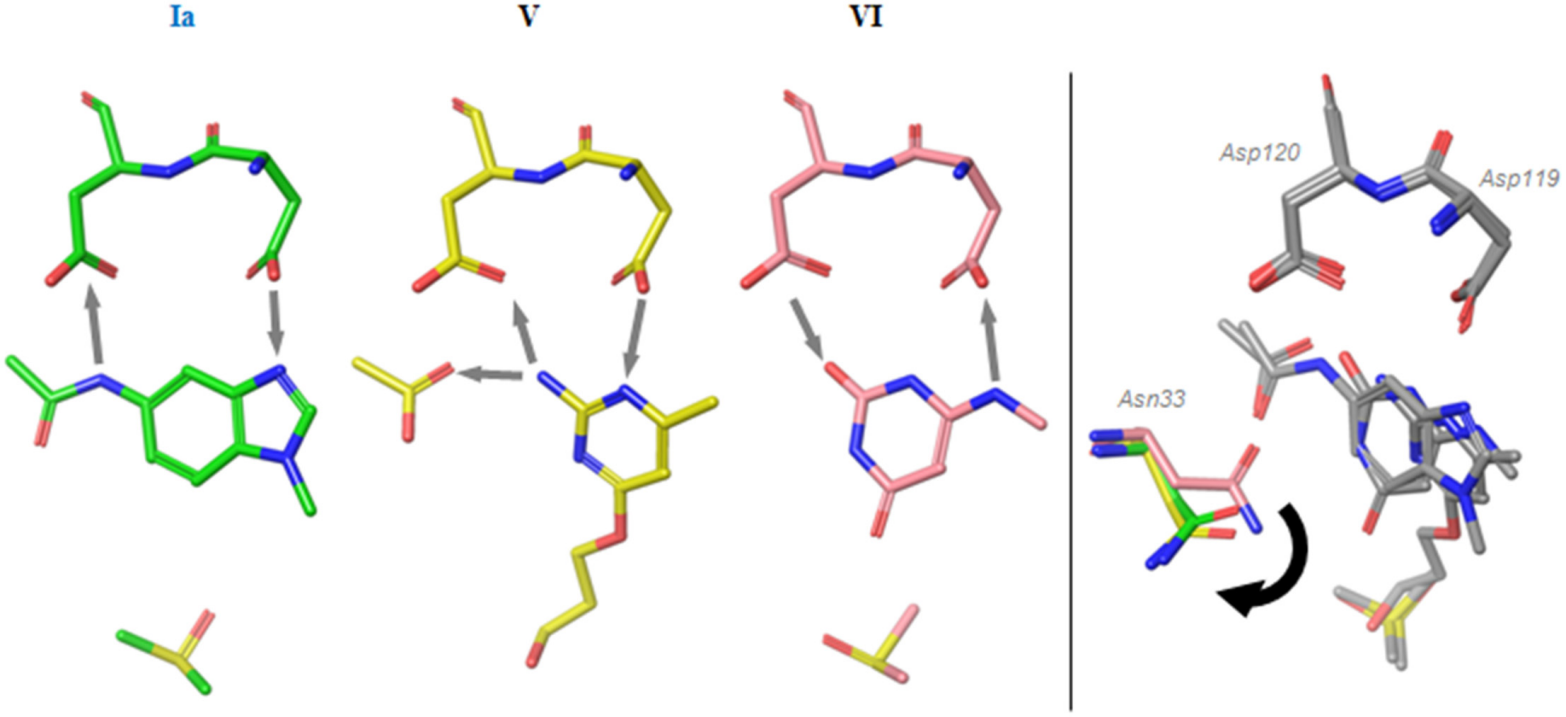
Fragment binding modes. a) binding modes of compounds 5, 6 and 7; b) movement of Asn33.

To further highlight the variability in the hydrogen bonding patterns seen with the Asp-Asp recognition element, we discuss here the structures obtained with compounds **5**, **6**, and **7**. Their binding modes are shown in Fig 7 and it is interesting to note that none of the fragment binding modes resemble the binding modes **II**, **III** and **IV** seen in Fig 2.

Fragment **5** (binding mode **Ia**) aligns well with the nucleobase of 8-oxo-dGTP (binding mode **I**) but lacks the central hydrogen bond donor seen in the oxidized base. The phenyl ring of the bicyclic system is however ideally placed for a favourable contact with the central carbonyls, eliminating the need for a hydrogen bond usually seen in this position. Additionally, it places an acetyl group binding in the location of the methyl group seen in binding mode **IV** (Fig 2), but unlike binding mode **IV**, its interactions span both of the residues in the Asp-Asp motif with a hydrogen bond donor and an acceptor.

The binding mode of fragment **6** exhibits incorporation of an acetate ion similar to that seen in the 8-oxo-dATP structure (Fig 4b) and matches its hydrogen bonding pattern. The flexible ether-linked chain partly occupies the ribose group pocket. The 4-methyl group of **6** displaces the same water that is affected by **3** (as shown in Fig 6), thereby stabilizing this binding mode in the absence of a bulky group in the ribose pocket.

The binding mode of fragment **7** (mode **VI**) exhibits a positioning of the Asn33 residue similar to that seen in the 8-oxo-dGTP nucleotide structure, whereas the residue is flipped out in the case of binding modes **Ia** and **V** (Fig 7b). In both cases this is the result of steric interactions and/or displacement of a bound water (not shown) by the acetyl group in the case of **Ia**, or the recruited acetate ion in the case of binding mode **V**. A similar movement of this Asn residue is also observed in the structure of **3**. The acidity of the carbonyl group of **7** (pK_a_ 4.1, calculated value) suggests that it can exist in the keto form, consistent with the expected keto form of an 8-oxoguanine base [33]. The distances to the aspartate oxygens (2.5 Å and 3.2 Å) of Asp120 suggest a strong hydrogen bond.

## Conclusions

**Fig 8:**
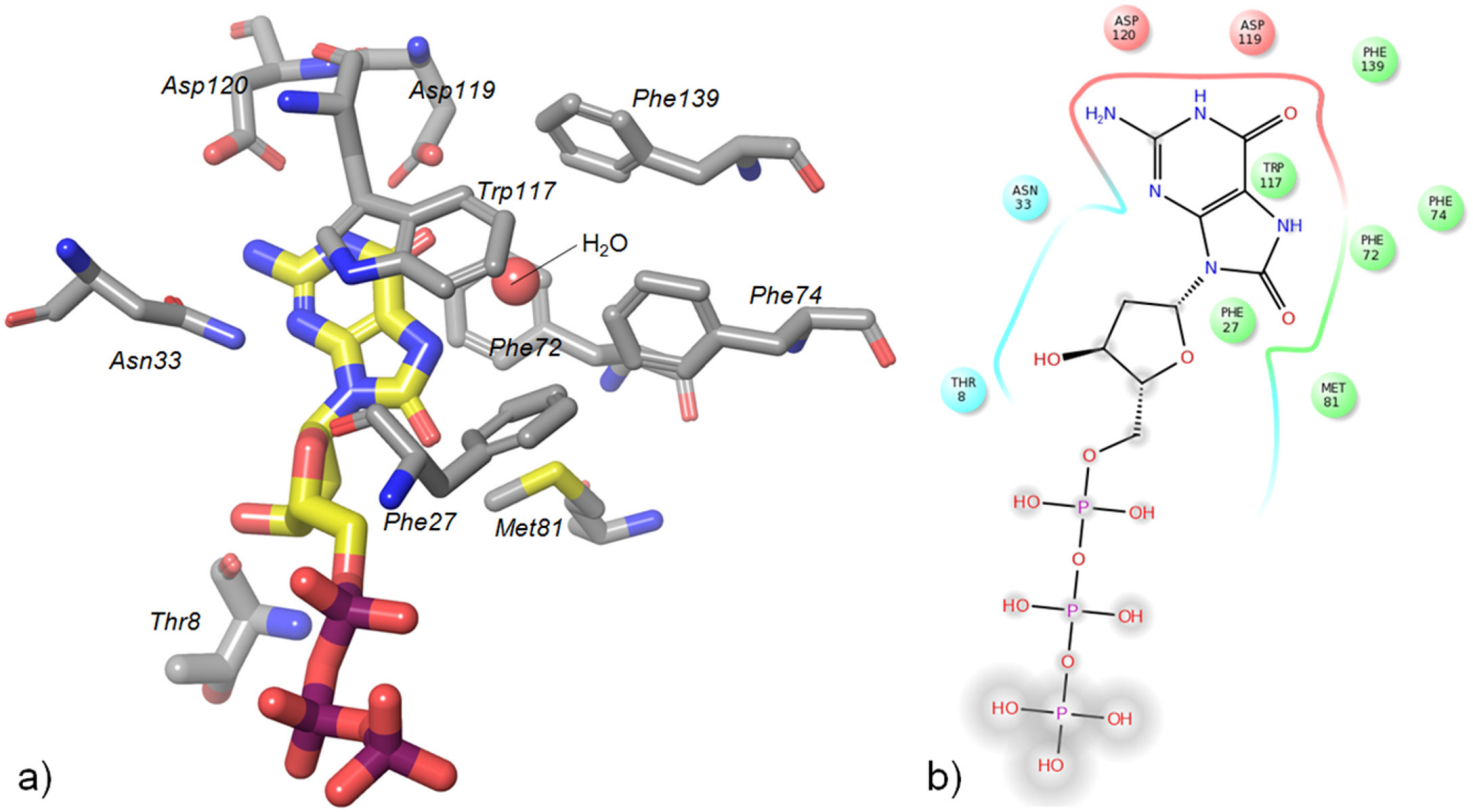
Key binding and recognition residues of MTH1. a) Binding site and b) schematic overview. The Asp-Asp motif, Met81, Trp117, Phe27 and Asn33 as well as the water play key roles in recognising and binding the nucleobase. Thr8 interacts with the ribose.

Interest in MTH1 as an oncology target has re-emerged recently and its inhibition has been suggested to be beneficial in a wide range of tumours.[7,8] We discuss here a series of substrate and fragment binding modes observed with the MTH1 protein in their protein structures. Fig 8 summarises the key binding and recognition interactions of the MTH1 pocket with its substrate. The variety of binding modes shown by ligands interacting with the Asp-Asp motif (as well as Asn33, Phe72 and Trp117) in the nucleotide binding pocket of MTH1 suggest that this key binding motif has evolved as a promiscuous binder of a variety of nucleobase-containing substrates. A threonine residue near the ribose sub-pocket has been suggested previously to contribute to recognition of the ribose group [6]. We show here that specific contacts with the substrate confer selectivity for oxidized nucleotides over their non-oxidised counterparts. These include favourable protein-substrate contacts with Met81 and Phe27, as well as coordination of a water positioned near the hydrophobic pocket formed by Phe72, 74 & 139 and Met81. We further demonstrate that the key anchoring element of MTH1 is its Asp-Asp motif, and that it recognises a wide range of hydrogen-bonding motifs. The binding motifs presented in this in this paper offer insights that may aid the development of specific and selective inhibitors of this cancer target.

## Supporting Information

S1 File Fig A. Calculated pKa values for nucleoside models (N-methylated nucleobase variants) suggest predominant tautomers. Compounds mimic a) guanine, b) 8-oxoguanine, c) 8-oxoadenine, and fragments d) **7** and e) **3**. Fig B. Image of the electron density of the substrate 8-oxo-dGTP. Density (2F_o_F_c_) is contoured at 1 σ. PDB reference 5FSL.
(DOCX)
