# Supplementary material for: MTH1 Substrate Recognition—An Example of Specific Promiscuity
Source: PLoS One. 2016 Mar 21;11(3):e0151154. doi: 10.1371/journal.pone.0151154 (PMC4801406; doi:10.1371/journal.pone.0151154)
Supplement: S1 File — Fig A. Calculated pKa values for nucleoside models (N-methylated nucleobase variants) suggest predominant tautomers. Compounds mimic a) guanine, b) 8-oxoguanine, c) 8-oxoadenine, and fragments d) 7 and e) 3. Fig B. Image of the electron density of the substrate 8-oxo-dGTP. Density (2FoFc) is contoured at 1 σ. PDB reference 5FSL. (DOCX) [file pone.0151154.s001.docx]

**Supporting Information** for Nissink et al., “MTH1 substrate recognition – an example of specific promiscuity”


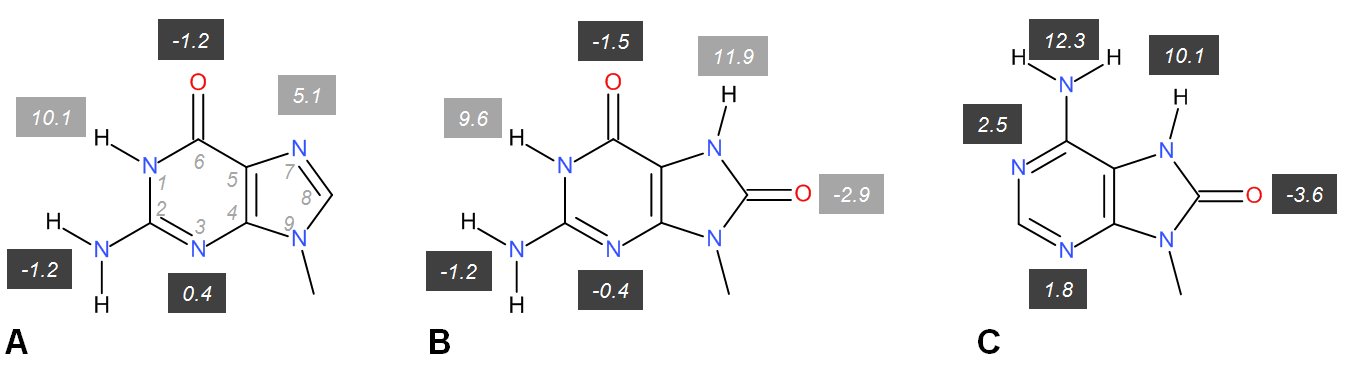


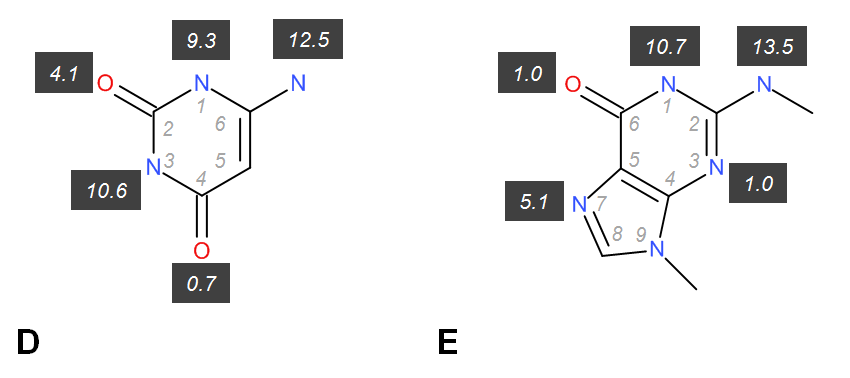


**Figure S1**. Calculated pKa values for nucleoside models (N-methylated nucleobase variants) suggest predominant tautomers. Compounds mimic a) guanine, b) 8-oxoguanine, c) 8-oxoadenine, and fragments d) **7** and e) **3**.
Calculations were performed in Maestro v10.1.012, Release 2015-1 (Schrodinger, LLC, New York, NY, 2015), using the quantummechanical pKa module (Jaguar, version 8.7, Schrodinger, LLC, New York, NY, 2015). Solvent was water.


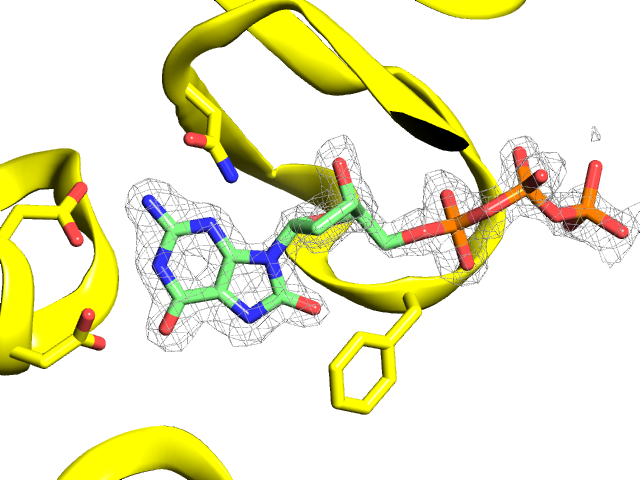


**Figure S2**. Image of the electron density of the substrate 8-oxo-dGTP. Density (2F_o_F_c_) is contoured at 1 σ. PDB reference 5FSL.
